# Supplementary material for: QoALa: A comprehensive workflow for viral quasispecies diversity comparison using long-read sequencing data
Source: PLoS Comput Biol. 2026 Apr 28;22(4):e1014208. doi: 10.1371/journal.pcbi.1014208 (PMC13123935; doi:10.1371/journal.pcbi.1014208)
Supplement: S1 Fig — Connected scatter plot with points indicating the percentage of singleton haplotypes (y-axis: pctsingleton) at different noise-minimization cut-off percentages (x-axis: pct). Lines and dots are colored by individual samples. Vertical dashed lines represent selected cut-off percentages for each gene and viral species. The high proportion of singleton haplotypes observed in some datasets (e.g., HCV NS4), despite being generated using high-accuracy PacBio CCS sequencing, likely reflects the combined effects of residual sequencing errors at the haplotype level and genuine within-host viral diversity, particularly in chronic infections such as HCV where even single-nucleotide differences can define distinct haplotypes. (DOCX) [file pcbi.1014208.s001.docx]

S1 Fig: Selection of noise-minimization’s cut-off percentage based on percentage of singleton haplotypes.


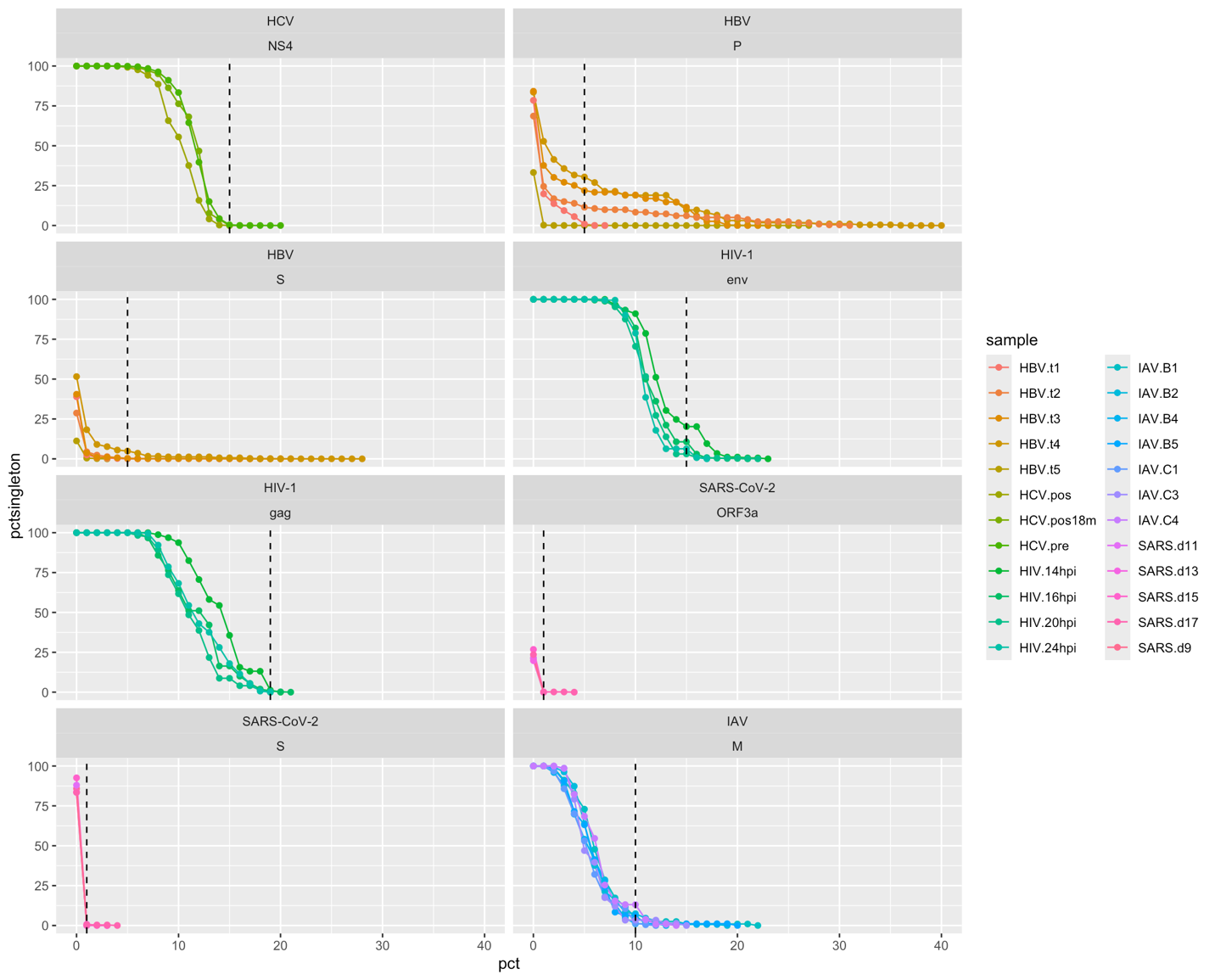


Connected scatter plot with points indicating the percentage of singleton haplotypes (y-axis: pctsingleton) changing at different noise-minimization cut-off percentages (x-axis: pct). Lines and dots are colored by individual samples. Vertical dashed lines represent selected cut-off percentages for each gene and viral species. The high proportion of singleton haplotypes observed in some datasets (e.g., HCV NS4), despite being generated using high-accuracy PacBio CCS sequencing, likely reflects the combined effects of residual sequencing errors at the haplotype level and genuine within-host viral diversity, particularly in chronic infections such as HCV where even single-nucleotide differences can define distinct haplotypes.
