# Supplementary material for: QoALa: A comprehensive workflow for viral quasispecies diversity comparison using long-read sequencing data
Source: PLoS Comput Biol. 2026 Apr 28;22(4):e1014208. doi: 10.1371/journal.pcbi.1014208 (PMC13123935; doi:10.1371/journal.pcbi.1014208)
Supplement: S10 Fig — Same layout as S2 Fig. (DOCX) [file pcbi.1014208.s010.docx]

S10 Fig: Effect of down-sampling after noise-minimization on nine diversity metrics in IAV’s M gene segment samples.
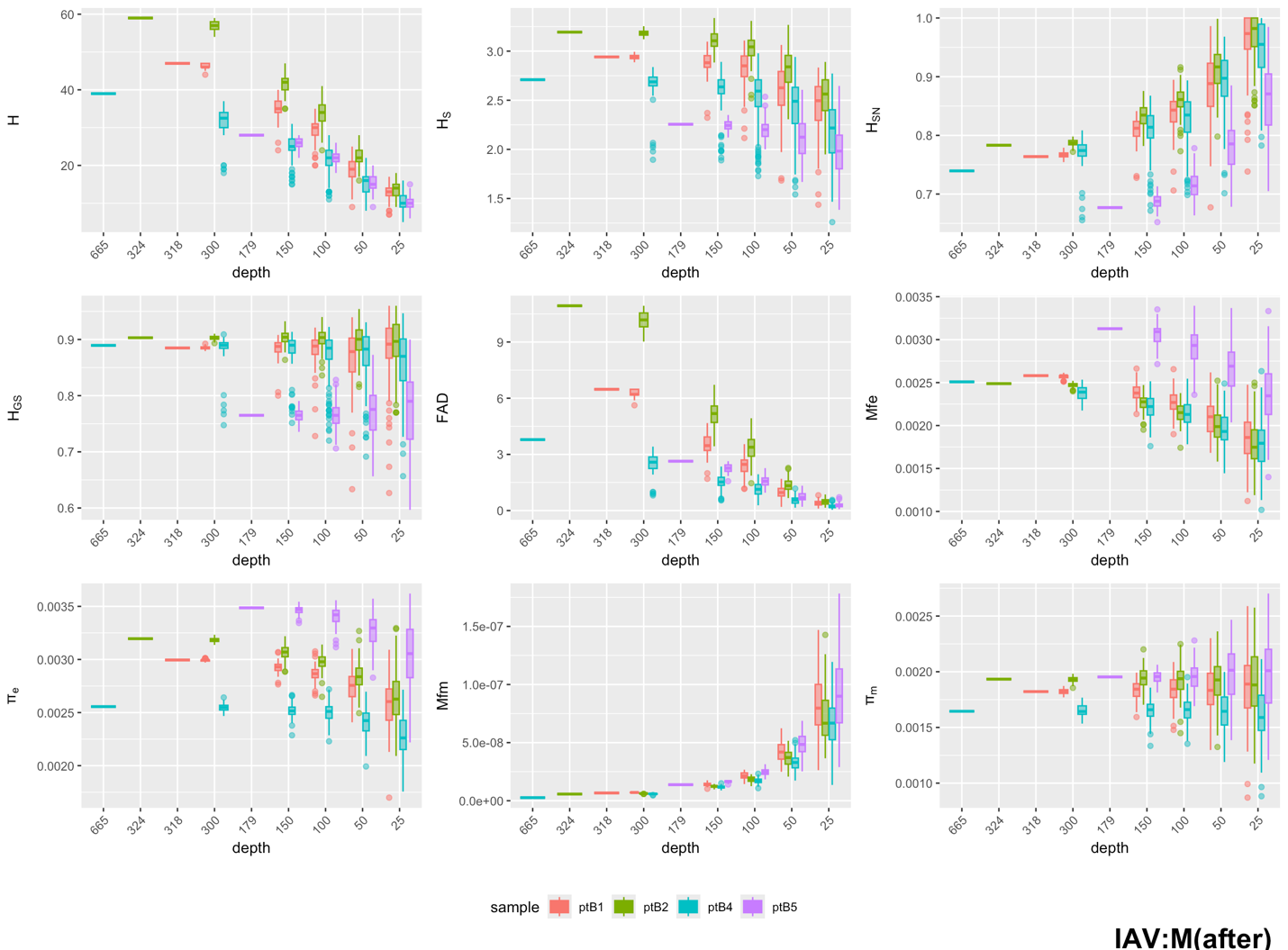


Distribution of nine diversity metrics, including the number of haplotypes (H), Shannon entropy (H_S_), normalized Shannon entropy (H_SN_), Gini-Simpson index (H_GS_), functional attribute diversity (FAD), mutation frequency at the entity level (Mfe), nucleotide diversity at the entity level (π_e_), mutation frequency at the molecular level (Mfm), and nucleotide diversity (π_m_). These metrics are computed at different sample sizes (100 repeated random samplings with replacement for each size), arranged from left to right and top to bottom. For all plots, horizontal lines indicate medians, boxes the 25th to 75th percentile, whiskers the lowest and highest values excluding outliers, and dots the outliers.
