# Supplementary material for: QoALa: A comprehensive workflow for viral quasispecies diversity comparison using long-read sequencing data
Source: PLoS Comput Biol. 2026 Apr 28;22(4):e1014208. doi: 10.1371/journal.pcbi.1014208 (PMC13123935; doi:10.1371/journal.pcbi.1014208)
Supplement: S12 Fig — From top to bottom: stacked bar chart of unique haplotype proportions; stacked bar chart of new OTUs; MDS plot of haplotypes’ pairwise SNV distance highlighting five largest OTUs; MDS plot of all OTUs. (DOCX) [file pcbi.1014208.s012.docx]

S12 Fig: Viral quasispecies and OTU comparisons among HBV’s *P* and *S* genes samples.


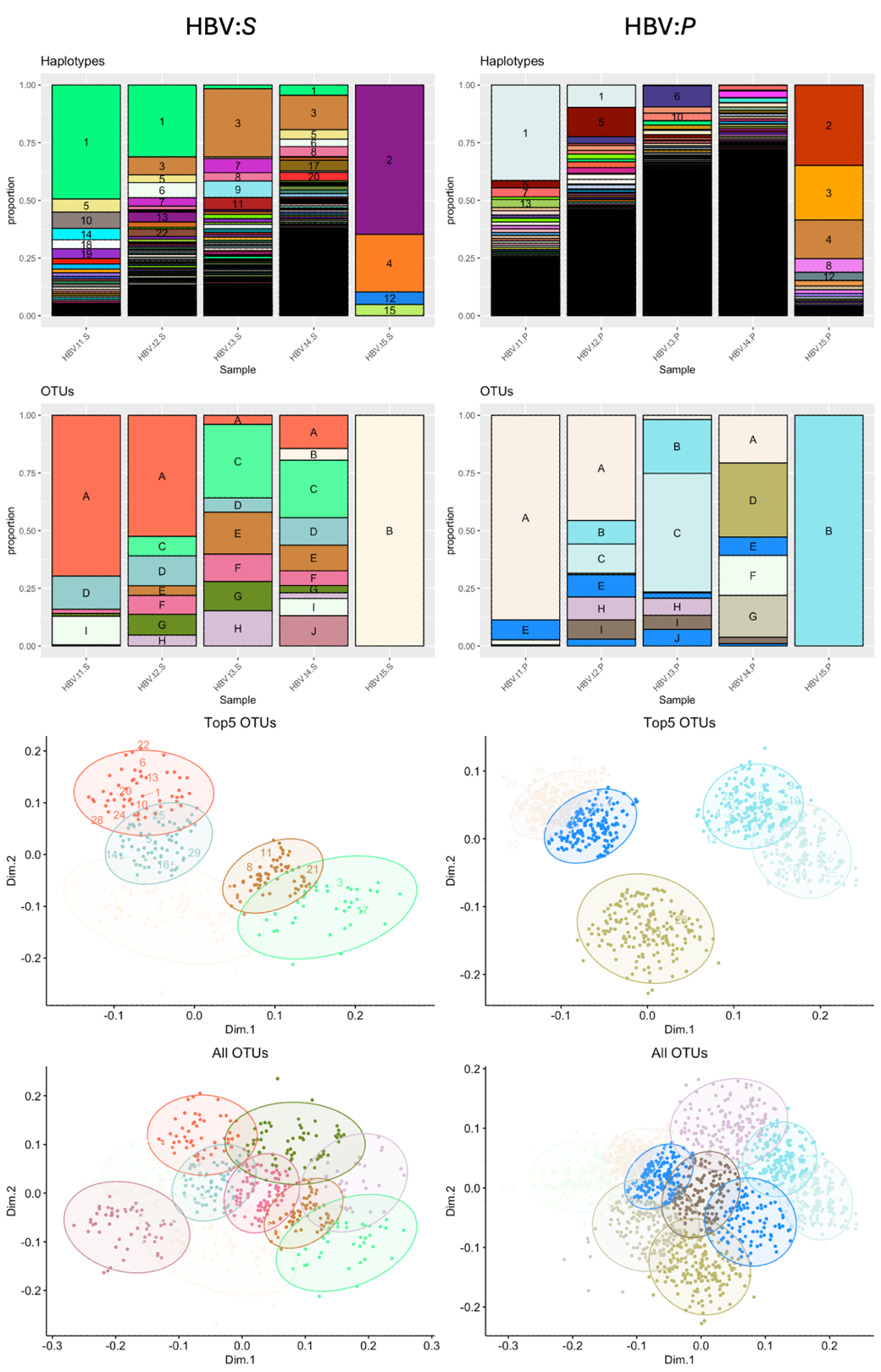


From top to bottom: 1. Stacked bar chart presenting the proportion of each unique haplotype (same color) identified across different samples within each gene dataset. 2. Stacked bar chart presenting the proportion of new OTUs, each consisting of genetically closely related haplotypes. 3. Multidimensional scaling (MDS) plot of haplotypes' pairwise SNV distance (dot), showing the five largest OTUs (circle) with a color scheme corresponding to plot b. 4. MDS plot of all OTUs.
