# Supplementary material for: QoALa: A comprehensive workflow for viral quasispecies diversity comparison using long-read sequencing data
Source: PLoS Comput Biol. 2026 Apr 28;22(4):e1014208. doi: 10.1371/journal.pcbi.1014208 (PMC13123935; doi:10.1371/journal.pcbi.1014208)
Supplement: S14 Fig — Same layout as S12 Fig. (DOCX) [file pcbi.1014208.s014.docx]

S14 Fig: Viral quasispecies and OTU comparisons among SARS-CoV-2’s *S* and *ORF3a* genes samples.


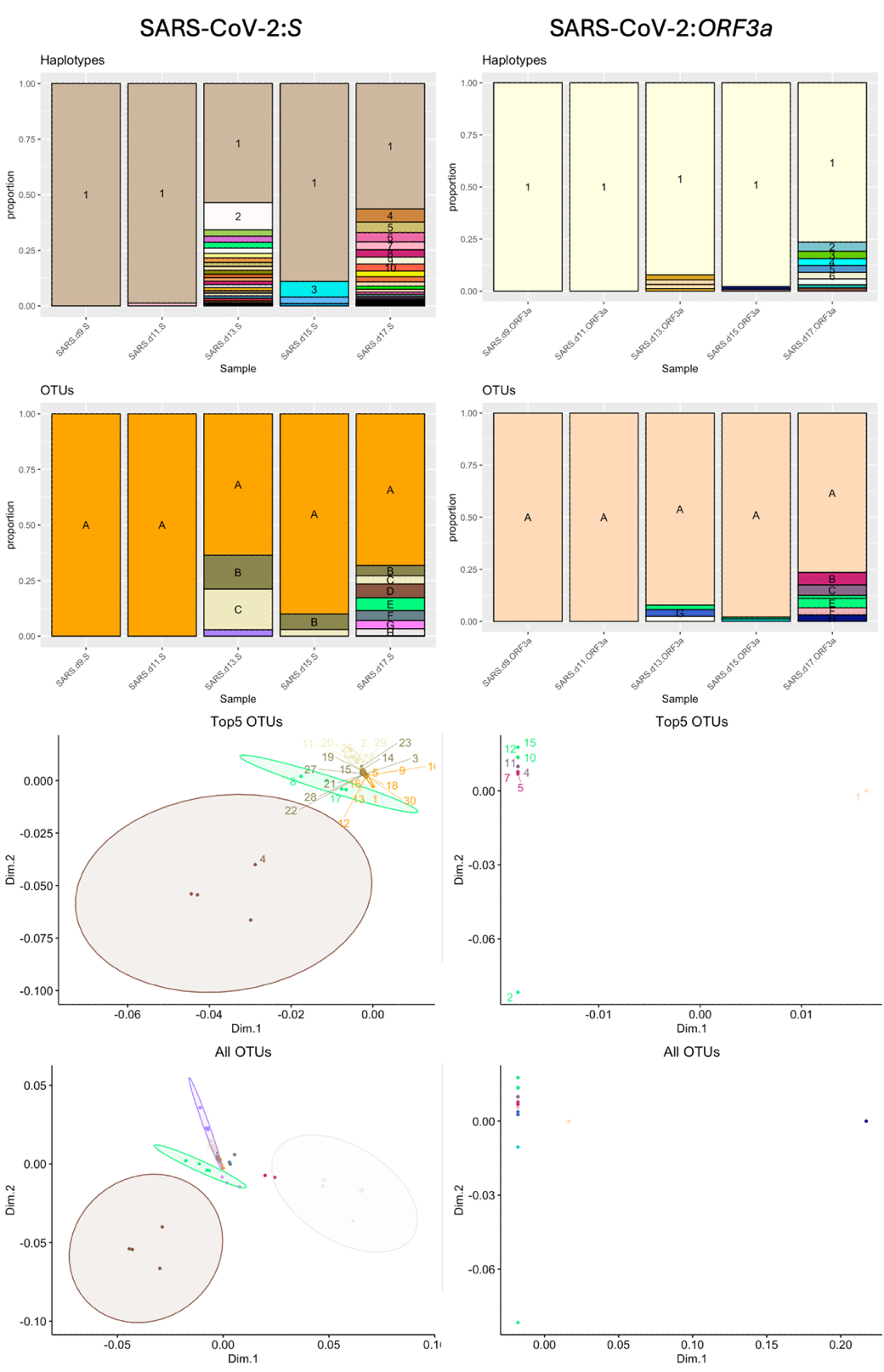


From top to bottom: 1. Stacked bar chart presenting the proportion of each unique haplotype (same color) identified across different samples within each gene dataset. 2. Stacked bar chart presenting the proportion of new OTUs, each consisting of genetically closely related haplotypes. 3. Multidimensional scaling (MDS) plot of haplotypes' pairwise SNV distance (dot), showing the five largest OTUs (circle) with a color scheme corresponding to plot b. 4. MDS plot of all OTUs.
