# Supplementary material for: QoALa: A comprehensive workflow for viral quasispecies diversity comparison using long-read sequencing data
Source: PLoS Comput Biol. 2026 Apr 28;22(4):e1014208. doi: 10.1371/journal.pcbi.1014208 (PMC13123935; doi:10.1371/journal.pcbi.1014208)
Supplement: S15 Fig — Connected scatter plots showing percentage of singleton haplotypes (y-axis) versus noise-minimization cut-off (x-axis). Lines and points colored by sample; vertical dashed lines indicate median cut-off for each sequencing platform and virus:gene combination. (DOCX) [file pcbi.1014208.s015.docx]

S15 Fig: Selection of noise-minimization cut-off percentage based on singleton haplotype frequency in simulated data


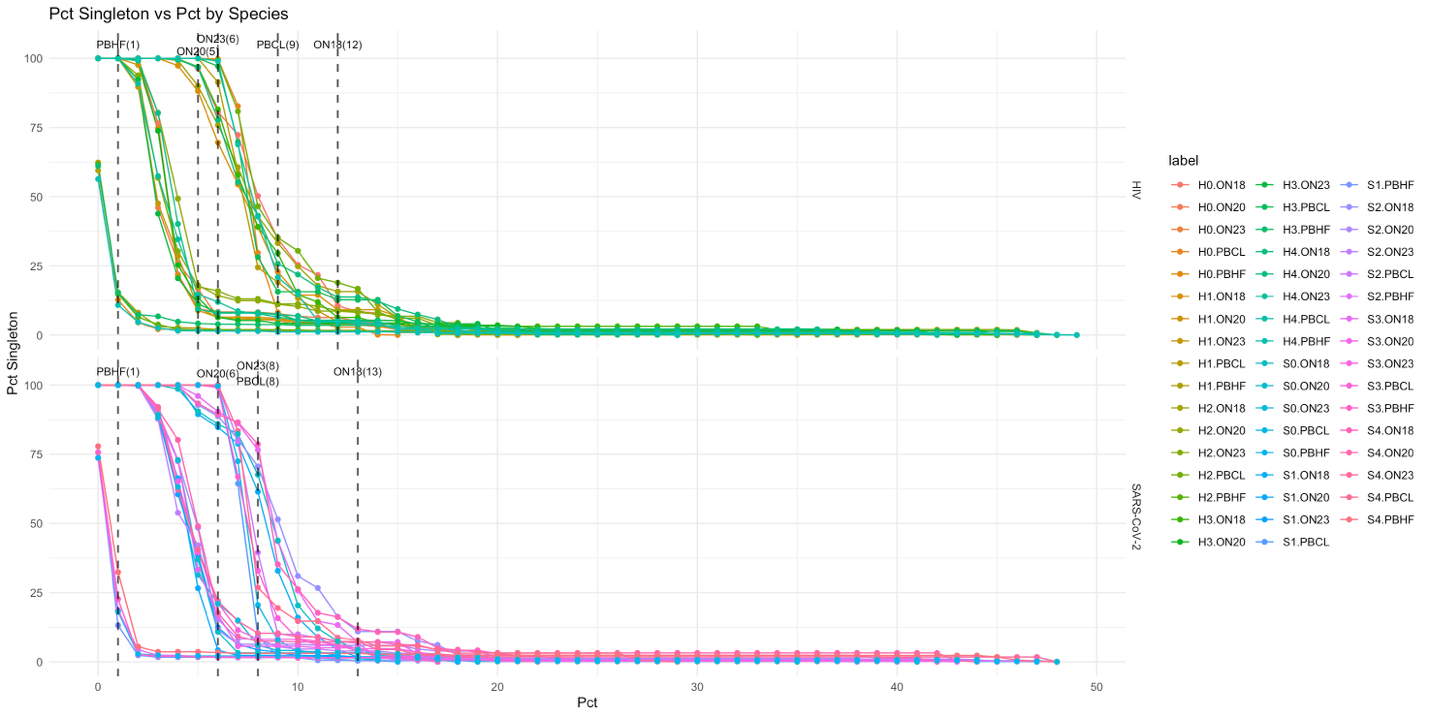


Connected scatter plots showing the percentage of singleton haplotypes (y-axis: Pct Singleton) as a function of noise-minimization cut-off percentage (x-axis: Pct). Lines and points are colored by individual samples, with each line representing the trajectory of singleton frequency across cut-off values for a given sample. Vertical dashed lines indicate the median cut-off percentage selected for each sequencing platform and virus:gene combination (top: HIV-1 *env*; bottom: SARS-CoV-2 *S*). Numbers in represent median values across replicates for each sequencing platform.
