# Supplementary material for: QoALa: A comprehensive workflow for viral quasispecies diversity comparison using long-read sequencing data
Source: PLoS Comput Biol. 2026 Apr 28;22(4):e1014208. doi: 10.1371/journal.pcbi.1014208 (PMC13123935; doi:10.1371/journal.pcbi.1014208)
Supplement: Attachment — Submitted filename: reviewing.pdf [file pcbi.1014208.s023.pdf]

Pamornchainavakul and co-authors developed a new tool, QoALa, to provide comparative results to investigate viral quasispecies in intra and inter-hosts dynamics with long-reads sequencing technology. This tool allows viral populations to be analyzed and visualized during longitudinal studies.
The article is well written, the context and the aims are well defined. The R package they developped, longreadvqs, is available on the CRAN.
The authors took care to evaluate the performance of their tools using simulated datasets as well as real datasets produced by various ONT and PacBio technologies.

Concerning the software, in order to facilitate the use and distribution of QoALa, it would be useful for the authors to provide a ready-to-use environment, in Conda or another format. Indeed, it can sometimes be complicated to meet all dependencies depending on the version of R used and the packages included in their longreadvqs package.

<u>Minor points to consider</u> :

1- Supplementary figure 1, for real data, it is surprising that the HCV dataset shows nearly 100% singletons on NS4 in the uncorrected reads, particularly given that PacBio CCS data are generally less error-prone than CLR or ONT data. In contrast, the high proportion of singletons observed for HIV-1 *env* and *gag*, as well as for IAV, is less unexpected in datasets generated with ONT. Do the authors have an explanation for this unusually high level of singletons observed in the PacBio CCS data?

2- Still referring to the real sequencing data, line 513, it is noted that 10% of the lowest quality reads are eliminated. Is this done even if the Qscore of these lowest quality reads exceeds Q30, for example? Wouldn't it be better to set a threshold at 10% **unless** the reads have a score of Qx?

3- Supplementary figure 1, for HIV-1 *env* and *gag*, how can we explain that when the cut-off value increases, the percentage of singletons also sometimes increases? An illustrative example: for HIV-1 *env*, with the HIV.14hpi sample, a cut-off of ~16% produces ~23% singletons, while a cut-off of ~17% produces ~27% singletons. Shouldn't the proportion of singletons always decrease as the cut-off percentage increases? Highlighted with the red circle on the figure :

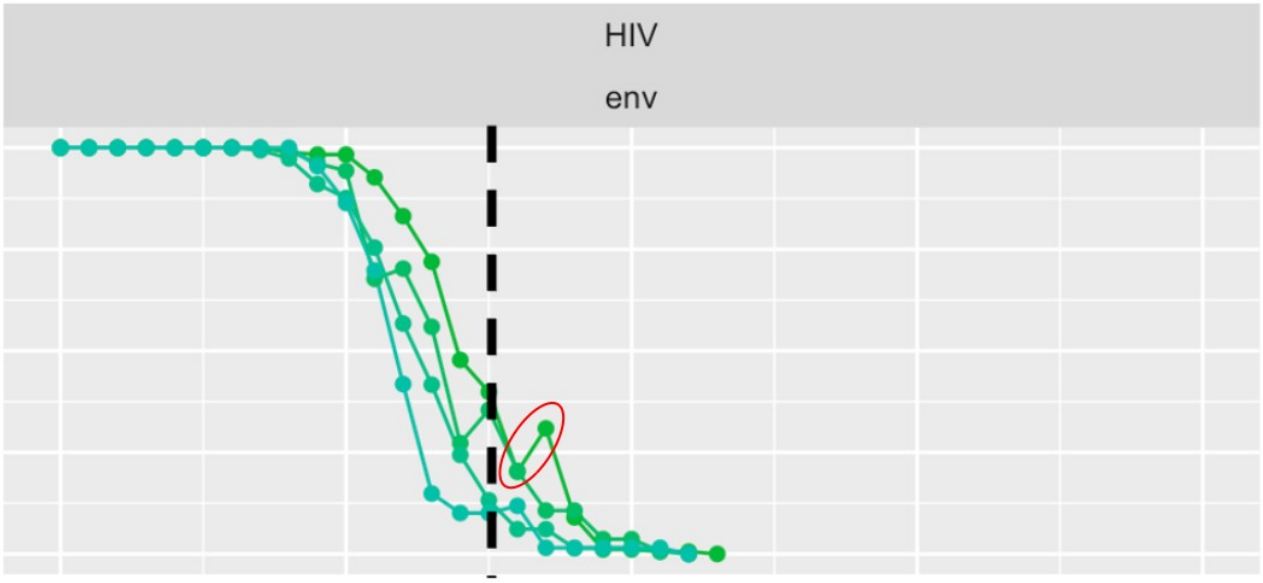

4- For the nucleotide position-based noise reduction method adopted by the authors, which corrects erroneous SNVs by assigning the majority base at each position, co-infection scenarios may introduce specific limitations. In particular, if a singleton read originating from infection 1 is corrected at a position where infection 2 is predominant, the correction may be erroneous. Conversely, co-infection may also reduce the number of corrections by decreasing the proportion of reads falling below the SNV frequency cutoff at a given position.

For example in a sample with a co-infection, at nucleotide position *n*, if a read from infection 1 carries a nucleotide *A* that is a sequencing error, while *A* is the genuine nucleotide at the same position in infection 2, the erroneous base in the read from infection 1 will not be corrected. This limitation is inherent to position-specific correction approaches, which rely on per-site nucleotide frequencies rather than on information from the full read or haplotype context.

While this does not constitute an inherent flaw, as it reflects a methodological choice, it may be worth discussing this point as a limitation of the approach.

5- The link to the simulated data in the GitHub repository, line 684, is invalid, although it is easy to find this data in the repository.

6- Specify HIV-1 when referring to HIV, as the authors distinguish between IAV and IBV.
