# Supplementary material for: QoALa: A comprehensive workflow for viral quasispecies diversity comparison using long-read sequencing data
Source: PLoS Comput Biol. 2026 Apr 28;22(4):e1014208. doi: 10.1371/journal.pcbi.1014208 (PMC13123935; doi:10.1371/journal.pcbi.1014208)
Supplement: Attachment — Submitted filename: Response to Reviewers.docx [file pcbi.1014208.s025.docx]

**Response to Reviewers**

Reviewer #1: Pamornchainavakul and co-authors developed a new tool, QoALa, to provide comparative results to investigate viral quasispecies in intra and inter-hosts dynamics with long-reads sequencing technology. This tool allows viral populations to be analyzed and visualized during longitudinal studies.

The article is well written, the context and the aims are well defined. The R package they developped, longreadvqs, is available on the CRAN. The authors took care to evaluate the performance of their tools using simulated datasets as well as real datasets produced by various ONT and PacBio technologies.

Concerning the software, in order to facilitate the use and distribution of QoALa, it would be useful for the authors to provide a ready-to-use environment, in Conda or another format. Indeed, it can sometimes be complicated to meet all dependencies depending on the version of R used and the packages included in their longreadvqs package.

**Response**: We thank the reviewer for this helpful suggestion regarding the usability and distribution of our package. The QoALa software was fully developed in the R programming language and is publicly available through CRAN. As a result, most required dependencies are automatically installed or updated when the package is installed. The only dependency that is not installed automatically is the QSutils package, which is distributed through Bioconductor and is installed via the devtools workflow rather than directly through CRAN. Dependencies installed from different repositories are common in both R and Python ecosystems, particularly when packages rely on specialized tools distributed through platforms such as Bioconductor or GitHub. To address this, we have provided clear instructions for installing this dependency separately in our GitHub repository (https://github.com/NakarinP/longreadvqs). This installation step is straightforward and only needs to be performed once during setup. As of March 5, 2026, all installation steps have been tested and function correctly. If installation or compatibility issues arise with newer versions of R or its dependencies, the CRAN maintenance system will notify us automatically, allowing us to promptly address and update the package. This has already occurred once when a function in one of the dependencies changed, and we resolved the issue accordingly. Once the required packages are installed, the working environment is effectively equivalent to a ready-to-use environment (similar to a Conda environment), except that it operates entirely within the R ecosystem rather than Python. We hope this clarification addresses the reviewer’s concern.

Minor points to consider :

1- Supplementary figure 1, for real data, it is surprising that the HCV dataset shows nearly 100% singletons on NS4 in the uncorrected reads, particularly given that PacBio CCS data are generally less error-prone than CLR or ONT data. In contrast, the high proportion of singletons observed for HIV-1 *env* and *gag*, as well as for IAV, is less unexpected in datasets generated with ONT. Do the authors have an explanation for this unusually high level of singletons observed in the PacBio CCS data?

**Response**: We thank the reviewer for this insightful observation. While PacBio CCS reads achieve high accuracy at the consensus genome level, this accuracy does not necessarily translate to haplotype-level resolution, where even a single nucleotide difference is sufficient to define a distinct haplotype. Residual sequencing errors in CCS reads — even at low per-base error rates — can therefore generate a substantial number of spurious singleton haplotypes.

Beyond sequencing noise, the gradual decline in singleton proportion observed for HCV NS4 (compared to the sharper drop seen in SARS-CoV-2 *S* gene samples that were also PacBio CCS reads; Supplementary Figure 1) suggests that biological diversity also contributes. HCV is known to form complex within-host quasispecies during chronic infection (https://doi.org/10.1016/0270-9139(95)90349-6), and the HCV dataset used here derives from chronic infection samples with documented extensive mutational diversity (https://doi.org/10.1038/s41598-022-11151-6). In contrast, the SARS-CoV-2 samples were collected over a short infection window (https://doi.org/10.1371/journal.ppat.1009431), reflecting more limited within-host diversity. This contrast is further supported by the HBV samples in our study, which were also sequenced by PacBio CCS and derived from chronic infection cases, yet exhibited low singleton proportions before noise minimization and a sharp decline afterward. This is consistent with the substantially lower evolutionary rate of HBV (~10⁻⁵ substitutions/site/year; https://doi.org/10.1128/jvi.00996-06) compared to both HCV and SARS-CoV-2 (~10⁻³ substitutions/site/year), resulting in limited within-host diversity despite the chronic infection context. The combination of residual CCS sequencing noise at the haplotype level and genuine biological diversity in chronic HCV infection thus likely explains the high singleton proportion and the need for a larger cutoff to reach the noise-minimization elbow point.

To clarify this point for readers, we have added a sentence to the Supplementary Figure 1: “*The high proportion of singleton haplotypes observed in some datasets (e.g., HCV NS4), despite being generated using high-accuracy PacBio CCS sequencing, likely reflects the combined effects of residual sequencing errors at the haplotype level and genuine within-host viral diversity, particularly in chronic infections such as HCV where even single-nucleotide differences can define distinct haplotypes.*”

2- Still referring to the real sequencing data, line 513, it is noted that 10% of the lowest quality reads are eliminated. Is this done even if the Qscore of these lowest quality reads exceeds Q30, for example? Wouldn't it be better to set a threshold at 10% **unless** the reads have a score of Qx?

**Response**: We thank the reviewer for this important point. To address this empirically, we analyzed the Q-score distributions of reads discarded by the --keep_percent 90 filter across all PacBio CCS samples. In all datasets, discarded reads had mean quality scores at or above Q30, confirming that the relative filter removed reads based on length rather than quality. This is particularly relevant in the context of viral quasispecies analysis, where full-gene read coverage is critical — retaining only reads of sufficient length ensures accurate haplotype reconstruction across the entire target region. We have updated the Methods section accordingly (Lines 560 to 565: “*Quality filtering was performed using Filtlong v0.2.1 to remove PacBio reads shorter than 1,000 bp or ranking in the lowest 10% by composite read score (a weighted combination of read length and mean quality); since all reads across datasets had mean quality scores at or above Q30, the relative filter removed reads based on length rather than quality. NanoFilt v2.8.0 was used to remove ONT reads with average quality scores < 7.*”).

3- Supplementary figure 1, for HIV-1 *env* and *gag*, how can we explain that when the cut-off value increases, the percentage of singletons also sometimes increases? An illustrative example: for HIV-1 *env*, with the HIV.14hpi sample, a cut-off of ~16% produces ~23% singletons, while a cut-off of ~17% produces ~27% singletons. Shouldn't the proportion of singletons always decrease as the cut-off percentage increases? Highlighted with the red circle on the figure :

**Response**: We thank the reviewer for this careful observation. Upon investigation, we identified two contributing factors explaining this non-monotonic behavior. First, the “*pctopt*” function had “*ignoregappositions = FALSE*” as its default, meaning gap characters were retained as variable sites during noise minimization — inconsistent with the downstream “*vqsassess*” function used for actual quasispecies analysis, which uses “*ignoregappositions = TRUE*”. Second, the HIV-1 *env* and *gag* alignments have relatively low read depth combined with gap-rich positions, making the dominant haplotype identity sensitive to small changes in the cut-off threshold. For example, at positions 462 and 1526 in the HIV-1 env alignment, gap (“-”) and nucleotide A occur at nearly equal frequencies (~58%/42% and ~51%/49% respectively). At cut-off 16%, the dominant haplotype carries A at both positions, so the “*CorrectGapsAndNs*” function of “*QSutils*” (part of both “*vqsassess”* and “*pctopt”* pipeline) — which corrects all gap/N positions in minority haplotypes to match the dominant haplotype after collapsing — unifies all haplotypes to A there, masking differences elsewhere and reducing distinct haplotypes. At cut-off 17%, a small shift in corrected reads causes the dominant haplotype to flip to - at both positions, reversing this unification and re-exposing previously masked differences — fragmenting merged haplotypes and transiently increasing singletons from 36 to 55 (~20% to ~31%). It is important to emphasize that this phenomenon is specific to alignments with abundant gap-rich positions at near-equal frequencies with nucleotide bases, as in the highly diverse HIV-1 *env* and *gag* genes. In the vast majority of samples in our study — where reads have low or no gap content — the noise minimization step effectively resolves sequence variation prior to haplotype collapsing, leaving “*CorrectGapsAndNs*” with no meaningful corrections to apply and producing stable, monotonically decreasing singleton curves as expected.

Setting “*ignoregappositions = TRUE*” resolves this entirely by uniformly masking gap-rich positions to - across all reads from the outset, stabilizing the dominant haplotype identity across all thresholds and producing the expected monotonically decreasing singleton curve. We have corrected this parameter inconsistency in “*pctopt*” and updated Supplementary Figure 1 accordingly. This adjustment did not affect the optimal cut-off for most genes, with the exception of HIV-1 *gag* where the elbow point shifted from 22% to 19%, for which we reran all downstream analyses and revised Supplementary Figure 13 accordingly.

4- For the nucleotide position-based noise reduction method adopted by the authors, which corrects erroneous SNVs by assigning the majority base at each position, co- infection scenarios may introduce specific limitations. In particular, if a singleton read originating from infection 1 is corrected at a position where infection 2 is predominant, the correction may be erroneous. Conversely, co-infection may also reduce the number of corrections by decreasing the proportion of reads falling below the SNV frequency cutoff at a given position.

For example in a sample with a co-infection, at nucleotide position *n*, if a read from infection 1 carries a nucleotide *A* that is a sequencing error, while *A* is the genuine nucleotide at the same position in infection 2, the erroneous base in the read from infection 1 will not be corrected. This limitation is inherent to position-specific correction approaches, which rely on per-site nucleotide frequencies rather than on information from the full read or haplotype context.

While this does not constitute an inherent flaw, as it reflects a methodological choice, it may be worth discussing this point as a limitation of the approach.

**Response**: We thank the reviewer for this insightful comment and fully agree that co-infection represents an important limitation worth discussing explicitly. We have added a dedicated paragraph to the Discussion section addressing this point (see below).

We agree that the “*conbase*” method (default) — which replaces low-frequency bases with the position-wise majority base — carries the risk described by the reviewer in co-infection scenarios, where a genuine minority-infection base may be incorrectly corrected toward the majority-infection consensus, or conversely where a sequencing error shared in frequency with a genuine variant from the co-infecting strain escapes correction. However, we would like to note that the severity of this limitation is relative to the alternative approaches. Our “*domhapbase*” method, which replaces low-frequency bases with the corresponding base of the single dominant haplotype regardless of position-wise frequency, carries a greater risk in co-infection scenarios — it would systematically pull all corrected reads toward the dominant infectious agent's sequence, potentially inflating its apparent quasispecies dominance more aggressively than “*conbase*”. More broadly, both methods are considerably more conservative than whole-haplotype replacement approaches, which would entirely eliminate minority haplotypes that could represent genuine variants from a co-infecting strain. We therefore maintain that “*conbase*” remains a reasonable methodological choice, while acknowledging that all frequency-based noise minimization approaches share the fundamental limitation that they cannot distinguish between sequencing error and genuine biological minority variants without additional contextual information such as linkage across the full read length.

We added this discussion in the following paragraph at Lines 473 to 487: “*However, it is worth noting that our position-wise noise minimization approach carries inherent limitations in co-infection scenarios. Because corrections are determined by per-position nucleotide frequencies across the entire read pool, a genuine minority-strain variant may be incorrectly corrected toward the majority-strain consensus, or a sequencing error may escape correction if it coincidentally matches a co-infecting strain's variant frequency. This limitation is less pronounced under “conbase” (the default method)—which replaces low-frequency bases with the position-wise majority base—than under “domhapbase”, which anchors all corrections to the single dominant haplotype and would more aggressively pull corrected reads toward the dominant strain at the expense of the minority. Both methods are nevertheless considerably more conservative than whole-haplotype replacement or removal approaches, which would eliminate minority haplotypes entirely. Consistent with this design, our benchmarking on simulated datasets showed that the approach preserved nucleotide diversity and the hierarchical population structure of quasispecies more accurately than alternative error-correction and haplotype reconstruction methods.”*

5- The link to the simulated data in the GitHub repository, line 684, is invalid, although it is easy to find this data in the repository.

**Response**: We thank the reviewer for spotting our broken hyperlink. We have corrected the hyperlink to <https://github.com/NakarinP/longreadvqs/tree/7184e665ee51b4e434d3b7b7c129d4d5745de1f7/sim_data> in the data availability statement (Lines 758 to 759).

6- Specify HIV-1 when referring to HIV, as the authors distinguish between IAV and IBV.

**Response**: We thank the reviewer for the concern on the virus nomenclature consistency. We have changed HIV to HIV-1 throughout the manuscript.

Reviewer #2: Pamornchainavakul et al develop workflow “QoALa” for viral quasispecies diversity comparison using long-read sequencing data. Evaluating the quasispecies composition is a challenging but an important task. The subject is interesting. The work is original and the visual presentation in the manuscript is impressive. However, there is a fundamental concern in the workflow: The noise-minimization step favors the variant with highest frequency at the position will skew the quasispecies profile.

**Response**: We thank the reviewer for raising this important concern regarding the position-wise noise-minimization step, which is a core component of our workflow prior to downstream analyses. We would like to clarify that this step—particularly the “*conbase*” method (our default approach in the QoALa workflow that replaces SNVs with frequencies below the cutoff with the consensus or majority nucleotide at that position)—is less likely to bias the quasispecies profile toward the dominant haplotype compared with several alternative strategies.

Specifically, approaches such as (1) “*domhapbase*” (our alternative method in the QoALa workflow that replaces low-frequency SNVs with the nucleotide present at the same position in the dominant haplotype), (2) replacing entire low-frequency haplotypes with the dominant haplotype, or (3) removing low-frequency or singleton haplotypes entirely (fringe trimming; <https://doi.org/10.1093/bioinformatics/btt768>), all directly increase the representation of the dominant haplotype. In contrast, the *conbase* method performs correction at the per-base level rather than the haplotype level. The nucleotide used for replacement is the majority base at that specific position across all reads, which does not necessarily correspond to the nucleotide present in the dominant haplotype. Consequently, this approach minimizes the likelihood of artificially increasing the frequency of any specific haplotype, including the dominant one.

We acknowledge that position-wise correction may alter the exact SNV composition of individual haplotypes, and therefore the corrected haplotypes may deviate from the true biological sequences. However, our objective in this step is to minimize sequencing noise while preserving the overall population structure used for downstream diversity comparisons. Compared with haplotype-level filtering or replacement approaches, the *conbase* method introduces the least bias in haplotype frequency distributions and therefore is less likely to substantially distort the inferred quasispecies profile. Importantly, this design choice is supported by our benchmarking on simulated datasets, where our method more accurately recovered the underlying quasispecies composition compared with existing error-correction and haplotype reconstruction tools such as Racon and Strainline, particularly in preserving nucleotide diversity and the hierarchical population structure (Fig. 2).

We have addressed our limitations highlighted by both reviewers in the following paragraph at Lines 473 to 487: “*However, it is worth noting that our position-wise noise minimization approach carries inherent limitations in co-infection scenarios. Because corrections are determined by per-position nucleotide frequencies across the entire read pool, a genuine minority-strain variant may be incorrectly corrected toward the majority-strain consensus, or a sequencing error may escape correction if it coincidentally matches a co-infecting strain's variant frequency. This limitation is less pronounced under “conbase” (the default method)—which replaces low-frequency bases with the position-wise majority base—than under “domhapbase”, which anchors all corrections to the single dominant haplotype and would more aggressively pull corrected reads toward the dominant strain at the expense of the minority. Both methods are nevertheless considerably more conservative than whole-haplotype replacement or removal approaches, which would eliminate minority haplotypes entirely. Consistent with this design, our benchmarking on simulated datasets showed that the approach preserved nucleotide diversity and the hierarchical population structure of quasispecies more accurately than alternative error-correction and haplotype reconstruction methods.”*

There are also some other concerns:

1. The workflow has been validated by simulation data and real Sample data. But validation results haven’t been presented. Therefore, we can’t evaluate.

**Response**: We thank the reviewer for raising this point. The QoALa workflow was validated using both simulated and real datasets, and these results are presented in the Results section. Specifically, benchmarking on simulated datasets across multiple sequencing platforms is shown in Fig. 2 and lines 198 to 241, where we evaluated the recovery of quasispecies composition and diversity using metrics including Bray–Curtis dissimilarity, Gini–Simpson diversity (H_GS_), and nucleotide diversity (π_m_). These analyses demonstrate that QoALa more accurately recovers the underlying quasispecies structure compared with alternative approaches. In addition, analyses on real datasets from multiple viruses (HIV-1, SARS-CoV-2, HCV, HBV, and IAV) are presented in Figs. 3–4 and lines 262 to 375, illustrating the robustness of diversity metrics and the ability of the workflow to capture biologically meaningful quasispecies dynamics across different scenarios. Additional results not shown in the main text are provided in the Supplementary Materials. To ensure transparency and reproducibility, all real sequencing datasets used in this study are publicly available through the NCBI Sequence Read Archive, and the simulated benchmarking datasets are also publicly accessible as described in the data availability statement. Furthermore, the full workflow is implemented in the *longreadvqs* package, which is publicly available via the Comprehensive R Archive Network.

2. The process of how the quasispecies sequence were obtained and how the proportion is calculated is not clear.

**Response**: We thank the reviewer for pointing out that the description of haplotype generation and frequency calculation was not sufficiently explicit. We have revised the Methods section to clarify how quasispecies sequences (haplotypes) are defined and how their proportions are calculated. Specifically, we added text explaining that haplotypes are defined as unique nucleotide sequences across the full-length alignment, that reads with identical sequences after noise minimization are grouped into the same haplotype, and that haplotype frequencies are calculated as the proportion of reads assigned to each haplotype relative to the total number of retained reads.

The following clarification has been added in the Methods section (lines 598–602): “*Haplotypes are defined as unique nucleotide sequences across the full-length alignment of the analyzed gene or genomic region. After noise minimization, reads with identical sequences are grouped into the same haplotype, and haplotype frequencies are calculated as the proportion of reads assigned to each haplotype relative to the total number of retained reads.*”

3. How the SNV profiles between samples are compared is not clear.

**Response**: We appreciate the reviewer’s suggestion and have clarified how SNV profiles are generated and compared between samples in the QoALa workflow. In the revised manuscript, we explicitly describe that SNV frequencies are summarized across alignment positions from the processed reads and that these SNV profiles are plotted and arranged across samples to enable visual comparison of variant patterns along the sequence.

The following clarification has been added in the Methods section (lines 603–607): “*For each sample, SNV frequencies across the alignment are summarized from the read data produced by ‘vqsassess’, and the resulting SNV frequency profiles are plotted and arranged across samples to enable visual comparison of variant patterns and positional consistency along the sequence (Fig. 1c).*”
